# Efficient *in vivo *knock-down of estrogen receptor alpha: application of recombinant adenovirus vectors for delivery of short hairpin RNA

**DOI:** 10.1186/1472-6750-6-11

**Published:** 2006-02-28

**Authors:** Yvonne D Krom, Frits J Fallaux, Ivo Que, Clemens Lowik, Ko Willems van Dijk

**Affiliations:** 1 Department of Human Genetics, Leiden University Medical Center, The Netherlands; 2 Netherlands Institute for Brain research, Amsterdam, The Netherlands; 3 Department of Endocrinology and Metabolism, Leiden University Medical Center, The Netherlands; 4 Department of General Internal Medicine, Leiden University Medical Center, The Netherlands

## Abstract

**Background:**

Adenovirus (Ad) mediated gene transfer is a well-established tool to transiently express constructs in livers of mice *in vivo*. In the present study, we determined the specificity and efficiency of Ad vectors expressing short hairpin (sh) RNA constructs to knock-down the estrogen receptor α (ERα).

**Results:**

Two different shRNA constructs derived from the murine ERα coding sequence were designed (shERα). *In vitro*, transfection of three mouse cell lines with pSUPER-shERα constructs resulted in up to 80% reduction of endogenous ERα activity. A single mismatch in the target sequence eliminated the reduction of ERα activity, demonstrating the specificity of shERα. The subsequently generated Ad.shERα vectors were equally effective *in vitro*. *In vivo*, intravenous administration of Ad.shERα resulted in 70% reduced hepatic mouse ERα mRNA levels. Co-injection of Ad.shERα with an Ad vector containing a luciferase (luc) gene driven by an estrogen responsive element (ERE) containing promoter resulted in a significant (90% on day five) down-regulation of hepatic luciferase activity, as determined by non-invasive optical imaging. Down-regulation was sustained up to day seven post-injection.

**Conclusion:**

Ad mediated transfer of shERα expression constructs results in efficient and specific knockdown of endogenous ERα transcription both *in vitro *and *in vivo*.

## Background

Estrogen exerts various biological effects in numerous organs throughout the body and has been implicated in the pathophysiology of a number of diseases including breast cancer, osteoporosis and cardiovascular disorders. Most of the estrogenic effects are mediated via the two known estrogen receptors, ERα and ERβ. These estrogen receptors are ligand-dependent transcription factors that can modulate gene transcription directly but also indirectly. Thus far, there is a relative paucity in the description of the role of estrogen and estrogen receptors in specific organs. Most studies have been performed using non-tissue specific manipulation of ER signaling such as complete knockouts either via deletion of the estrogen receptor or via deletion of estrogen production by ovariectomy. The availability of tools to specifically address the role of ER signaling in individual tissues would thus fill a void.

Short synthetic duplexes of 21 nucleotides long RNA molecules can specifically inhibit gene expression in mammalian cells [1]. Because of their efficacy and specificity, siRNA molecules provide a powerful tool to dissect gene function. To expand the applicability of the siRNA approach, Brummelkamp and co-workers [2] have introduced vector-based siRNA expression systems. By directing the synthesis of shRNA via the polymerase-III H1 RNA gene promoter, effective siRNA molecules are formed intracellular after transfection of shRNA expression constructs. To further expand the applicability of the siRNA approach, recombinant retro- and adenoviral based vectors have been designed [3,4]. Of these, adenoviral vectors offer the advantage of highly efficient infection of a broad range of cells, independent of active cell division. Moreover, high titers can be obtained and intravenous injection results in efficient transduction of the liver.

The present study was designed to generate tools to address the role of ERα in a tissue- and time- specific manner. To this end, we have developed recombinant Ad vectors encoding shRNA's directed against mouse ERα (Ad.shERα). Introduction of shERα, either by transfection or by Ad mediated gene transfer into different murine cell lines, led to efficient sequence specific repression of ER mediated transcription. Furthermore, intravenously administration of Ad.shERα resulted in efficient reduction of hepatic ERα mRNA levels (P < 0.005) and ERα functionality.

## Results

### Efficient and specific knock-down of endogenous mERα *in vitro*: Transfection with pSUPER-shERα constructs

Three pSUPER-derived vectors [2] designed to drive expression shERα sequences were constructed. Two vectors contained sh sequences derived from the boundary of the DNA binding domain and the hinge region (shERα_1103), or from the ligand binding domain (shERα_1395) of mERα, respectively. A third expression vector contained both the shERα_1103 and shERα_1395 expression cassettes in series (shERα_tandem).

**Figure 1:**
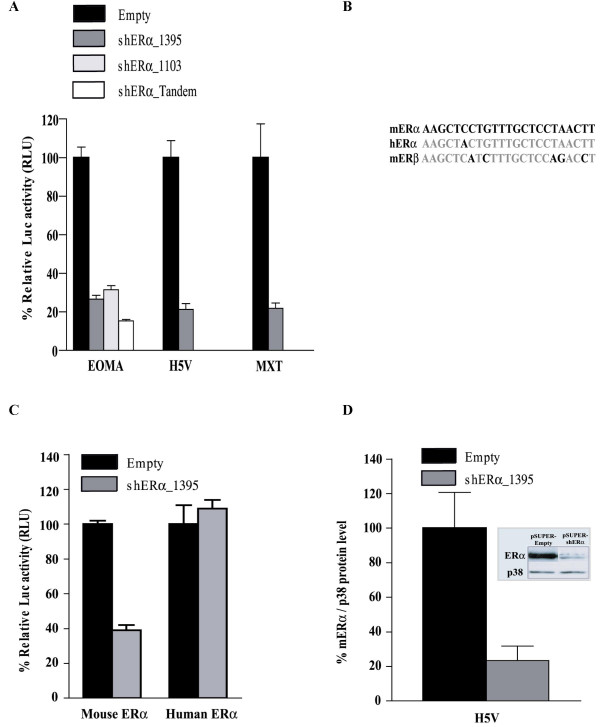
**Affectivity and specificity of pSUPER mediated expression of shERα in mouse cell lines**. (A+C) The indicated mouse cell lines were co-transfected with, ERE-Luc, CMV-LacZ, and pSUPER-empty, pSUPER-shERα_1395, pSUPER- shERα_1103, or pSUPER- shERα_tandem. Subsequently, the cells were treated 24 hours with 10^-9 ^M 17-β-estradiol. Luciferase activity was measured 48 hours after transfection and after correction for LacZ expression, represented as the mean (n = 3) ± SD relative to the transfection with pSUPER-empty. (A) Endogenous mouse ERα mediated transcription in EOMA, H5V and MXT cells after introducing pSUPER +/- shERα. (B) The 19-nt target-recognition sequence of ERα_1395 contains one mismatch with human ERα and five mismatches with the mouse ERβ sequence. (C) ERα mediated transcription in EOMA cells after over expression of either mouse ERα- or human ERα-expression vectors in presence of pSUPER empty or pSUPER shERα_1395 (D) Western blot analysis of H5V cells co-transfected with pCMV-mERα and pSUPER-empty or pSUPER-shERα_1395. The lysates were analysed by immunoblotting (insert-photo) with anti-mouse ERα and anti-p38. The intensity of the bands was quantified and normalized to cells transfected with pSUPER-empty. The relative ERα protein levels are presented (bar-diagram) as mean (n = 3) +/- SD.

The efficiency of the shERα constructs for reducing endogenous ERα activity *in vitro *was determined using a luciferase reporter assay. For this purpose, the pSUPER-shERα_1395, pSUPER-shERα_1103, or pSUPER-shERα_tandem were transfected together with a reporter plasmid carrying a trimer of ERE plus TATA box upstream of luciferase (pERE-Luc) into endothelial cell lines (EOMA and H5V) and in mouse breast cancer cells (MXT). As shown in Figure 1A, upon transfection with shERα_1395, relative luciferase activity in lysates of all three cell lines was reduced by 70–80%. A similar result was obtained with shERα_1103 in EOMA's. In addition, the shERα_tandem expression construct proved to be more efficient than either of single shERα contructs alone in the EOMA cells, adding some 15% to the 70% reduction observed with shERα_1395 (Fig. 1A).

To evaluate the specificity of the shERα construct, shERα_1395 was introduced into EOMA cells over-expressing either mouse ERα or human ERα. The ERα_1395 target sequence contains only a single mismatch with the human ERα (Fig. 1B). Significant suppression of ERα mediated transcription was only observed in lysates of cells that were transfected with mouse ERα but not with human ERα (Fig. 1C). Thus, the observed effects of shERα_1395 are specific for mouse ERα. Moreover, changing a single nucleotide in shERα_1395 completely abolished the silencing effect (data not shown). By western blotting, the effect of shERα on ERα protein expression was studied (Fig. 1D). In the presence of shERα_1395, ERα protein levels were reduced to 33% as compared to control transfected cells. This reduction correlated well with our findings in the luciferase reporter assay. Thus, the observed inhibition of luciferase activity upon treatment with shERα_1395 or shERα_1103 is caused by reduced accumulation of mERα protein. All together, the shERα_1395 and shERα_1103 expression vectors are effective and specific in repression of murine ERα expression.

### Knock-down of hepatic ERα expression *in vivo*: using Ad.shERα vectors

**Figure 2:**
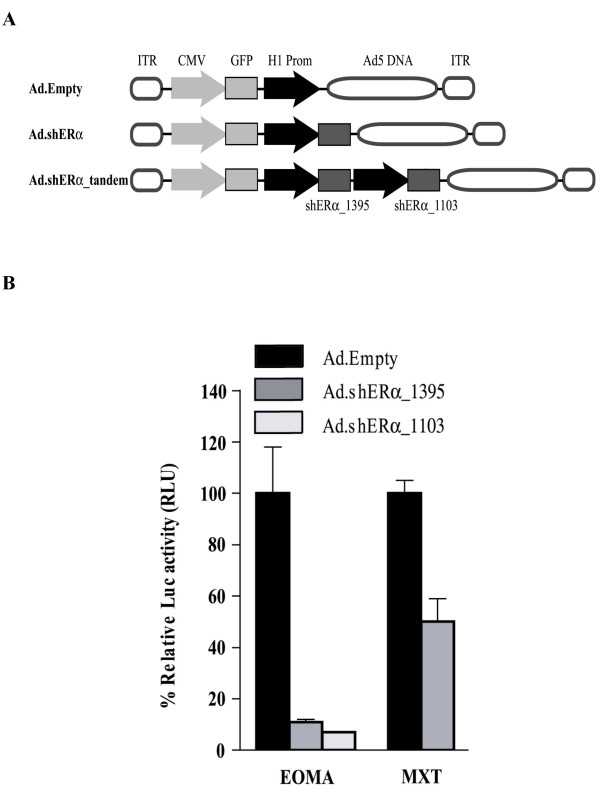
**ER-mediated luciferase activity after Ad-mediated transfer of shERα *in vitro***. (A) Schematic representation of the recombinant Ad vectors, carrying GFP and shERα expression cassettes that were used in this study. (B) EOMA and MXT cells were co-transfected with pERE-Luc and pCMV-LacZ and than infected either with Ad.Empty, Ad.shERα_1395, or Ad.shERα_1103. 10^-9 ^M Estrogen was administrated for 24 hours. Luciferase activity was measured 48 hours after infection. Data represented as mean ± SD relative to infection with Ad.Empty.

To repress ERα activity *in vivo*, Ad vectors expressing either shERα_1395 (Ad.shERα_1395), shERα_1103 (Ad.shERα_1103) or both (Ad.shERα_tandem) were generated (Fig. 2A). The H1 RNA promoter plus shERα expression cassettes were sub-cloned from the corresponding pSUPER into pAdTrack [5], which is engineered to co-express GFP enabling the tracking of infected cells. In addition, we constructed a control AdTrack plasmid, carrying only the H1 RNA promoter, which allowed for the generation of Ad.Empty. Prior to the evaluation of recombinant Ad vectors *in *vivo, we tested the functionality of the vectors *in *vitro. EOMA and MXT cells were transfected with pERE-luc, and subsequently infected with Ad.Empty or the Ad.shERα vectors. Fluorescence analysis indicated a near 100% infection percentage. The luciferase experiments (Fig. 2B) were comparable to those obtained with transfection of the pSUPER constructs (Fig. 1A): both Ad.shERα vectors repressed luciferase reporter activity up to 90%. Thus, Ad.shERα vectors were found to be fully functional with respect to repression of mERα activity.

**Figure 3:**
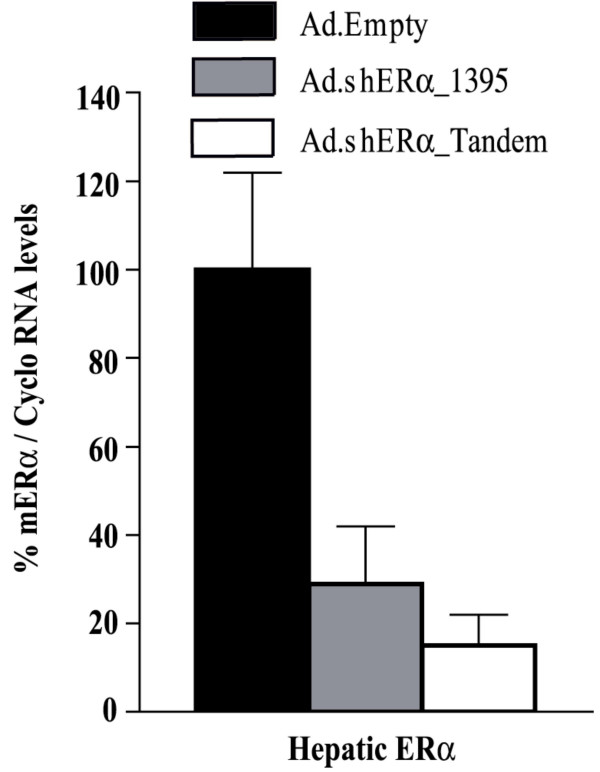
**Hepatic ERα mRNA levels after Ad-mediated transfer of shERα *in vivo***. Male C57Bl/6 mice (n = 5) were injected with 4 × 10^9 ^pfu Ad.Empty, Ad.shERα_1395 or Ad.shERα_tandem. Livers were harvested Four days after Ad. administration and subjected to taqman analysis. The cyclophillin gene was used as internal standard. Data represented as mean ± SD.

We then proceeded with the application of our vectors *in vivo*. The Ad vectors (Ad.Empty, Ad.shERα_1395, or Ad.shERα_tandem) were injected in the tail vain of C57Bl/6 mice. This allowed examination of inhibition by shERα of endogenous hepatic mERα. Four days post-injection, animals were sacrificed, and the livers were studied for GFP expression and ERα mRNA level. Similar GFP expression patterns were observed in all groups, indicating equally efficient transduction (data not shown). ERα mRNA levels were studied by real time PCR analysis (Fig. 3). Administration of Ad.shERα_1395 reduced ERα mRNA levels 70%, whereas hepatic expression of shERα_tandem resulted in an 85% reduction.

**Figure 4:**
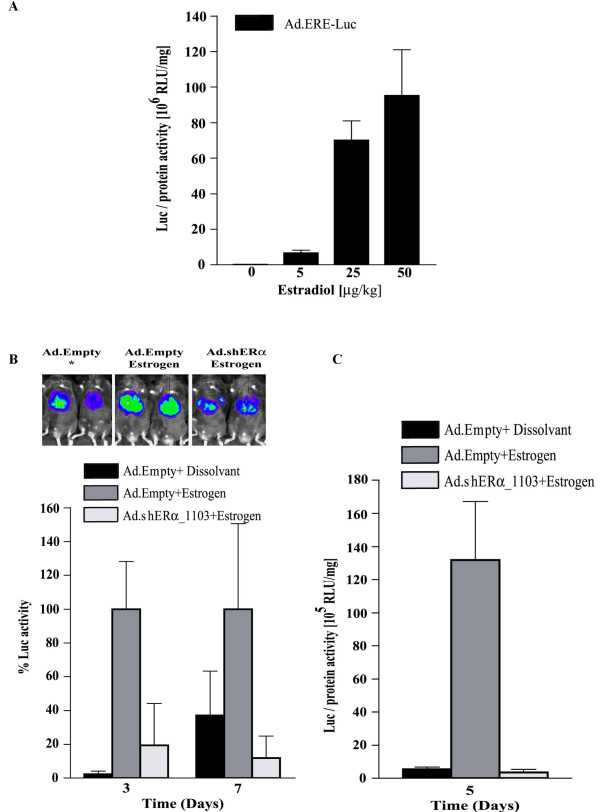
**Hepatic ERα activity**. (A) Male wt/ C57Bl/6 mice, pre injected with 5 × 10^8 ^pfu Ad.LacZ, were injected with 8 × 10^8 ^pfu Ad.ERELuc. Five days later, the recipients were treated for 6 hours with increasing amounts of estrogen (0–50 μg/kg, s.c). Then, the mice were sacrificed, and the livers were processed for luciferase assays. Luciferase activity is expressed as relative luciferase units (RLU) per mg total liver protein. (B) Male C57Bl/6 mice (n = 5) were injected with Ad.ERE-Luc (5 × 10^8 ^pfu) plus Ad.Empty or Ad.shERα_1103 (3 × 10^9 ^pfu). Three or seven days post-infection, the mice were injected with 5 μg/kg estrogen. The (inset) photo shows the result of optical imaging of the bioluminescence at day three, the bar-diagram is a quantitative representation of hepatic luciferase activity at day three or day seven. (C) Male C57Bl/6 mice (n = 5) were co-injected with Ad.ERELuc (5 × 10^8 ^pfu) + Ad.Empty or Ad.shERα_1103 (3 × 10^9 ^pfu). Five days later, the mice received 0 or 5 μg/kg estrogen. After 6 hours, the animals were sacrificed, and hepatic luciferase activity was determined. Luciferase activity is expressed as relative luciferase units (RLU) per mg total liver protein. Data represented as mean ± SD.

Subsequently, we sought to examine the extent of shERα-mediated repression of hepatic mERα transcription activity. For this purpose, we constructed an Ad vector carrying the estrogen responsive luciferase reporter gene (Ad.ERE-Luc). First the estrogen-responsiveness of this vector was determined *in vivo *(Fig. 4A). Five days post-injection of 8 × 10^8 ^pfu Ad.ERE-Luc, the mice were injected s.c with increasing concentrations of estrogen, ranging from 0 to 50 μg/kg. As shown in Fig 6, 6 hours post-injection, estrogen induced hepatic luciferase activity in a dose-dependent manner. Maximal stimulation was reached after applying 25 μg/kg estrogen. Then, we determined to what extend Ad.shERα down-regulates the transcriptional activity of hepatic ERα. Ad.shERα together with Ad.ERE-Luc reporter vector was administrated intravenously to C57BI/6 mice. Luciferase expression was detected by a CCD camera in living mice. Without estrogen treatment, all mice exhibited the same basal expression of the reporter construct (data not shown). Administration of 5 μg/kg estrogen, three and seven days after transduction with Ad.shERα_1103, resulted in a significant repression of hepatic ERα-mediated luciferase activity (Fig. 4B). These data were confirmed by measuring luciferase activity in liver extracts of mice that received estrogen (5 μg/kg, sc) five days post-injection with Ad.ERE-Luc plus Ad.Empty or Ad.shERα_1103 (Fig. 4C).

We conclude that Ad-mediated introduction of shERα *in vivo *results in an almost complete repression of hepatic mERα mRNA levels, as well as mERα-mediated transcription activity.

## Discussion

In this paper, we demonstrate that efficient silencing of mouse ERα can be achieved *in vitro *as well as *in vivo *by use of Ad-mediated transfer of shRNA molecules that target the ERα mRNA. Two independent shERα plasmid and Ad vector expression constructs were generated and shown to be effective in repressing endogenous ERα activity up to 80% in several different cell lines and *in vivo *(Fig. 1A, 2B and 3). In addition, a construct was made expressing both shERα sequences simultaneously. *In vitro *as well as *in vivo, *this construct was shown to be more effective (Fig. 1A and 3) than either of the two shERα constructs alone. Non-invasive optical imaging of living mice, allowed us to quantify shERα activity *in vivo. *Significant reduction of mouse ERα transcription levels were observed up to seven days post-transduction (Fig. 4B).

Thus far, bystander effects caused by shRNA constructs targeted to an unrelated gene have not been reported, and the specificity of the shERα_1395 construct was verified by the observation that human ERα, which has a single mismatch with the murine ERα target sequence, is not down-regulated (Fig. 1C). The number of mismatches with the murine ERβ sequence totals five, making it unlikely that the shERα_1395 construct would affect expression of ERβ. Similarly, the shERα_1103 construct has three mismatches with the human ERα and nine mismatches with murine ERβ, making it unlikely that the shERα_1103 construct would interfere with either of them. A single mismatch in the shERα_1395 sequence did render the construct ineffective in down-regulating murine ERα (data not shown). Thus, the two independent shERα constructs described here are exquisitely suited to demonstrate that a specific effect is mediated by down-regulation of ERα expression and not by down-regulation of a related sequence.

A key challenge in the application of an shRNA based approach is efficient delivery of the shRNA constructs to target cells *in vitro *and *in vivo*. For application of shRNA *in vivo*, the sh oligopair, driven by H1 RNA polymerase [2] or U6 promoter [6], can be cloned in viral vectors. Here, the Ad vector was chosen as delivery vector, because of the relative ease of generation and amplification. Moreover, the natural tropism of Ad vectors for the liver enables the rapid analysis of the hepatic knock-down phenotype. Since Ad vectors predominantly infect the parenchymal cells [7,8], it is important to note that most abundant hepatic ERα expression was detected in parenchymal cells while ERα expression was barely detected in hepatic endothelial cells or kupffer cells (data not shown). This supported the rationale for application of shERα Ad vectors *in vivo*. Another interesting observation was that upon administration of 4 × 10^9 ^pfu Ad.shERα, an 85% reduction of ERα mRNA levels was obtained (Fig. 5), whereas co-injection of 3 × 10^9 ^pfu Ad.shERα_1103 with 5 × 10^8 ^pfu Ad.ERE-Luc resulted in an almost complete absence of luciferase activity (Fig. 4C). The ratio of Ad.ERE-Luc *versus *Ad.shERα_1103 (1:6) should ensure that all cells that were transduced by Ad.ERE-Luc also received Ad.shERα_1103. Thus, the remainder of ERα expression determined by real-time PCR likely reflects ERα expression in non-parenchymal and non-infected cells.

Thus far, relative few reports describe the application of Ad vectors as delivery system for RNAi *in vitro *[9-12]. Similarly, relative few studies on effective RNA interference *in vivo *using Ad mediated gene transfer have been reported [13-15]. One potential explanation for this relative paucity in the application of Ad mediated gene transfer for shRNA expression constructs could lie in the recent observations of Lu and Cullen [16], that VA1 non-coding RNA, expressed by wild type adenovirus is a potent inhibitor of RNA interference. However, replication-incompetent adenovirus vectors such as the vectors used in our study have been reported to express low levels of VA1 [16]. Moreover, in our hands the effect of the pSUPER shRNA construct shERα_1935 on reduction of ERα activity in vitro was not affected by super-infection with the Ad.empty vector (data not shown). Thus, the Ad vectors applied in this study seem to have no or a minor inhibitory effect on the RNAi response in vitro and in vivo. Whether this effect is also insert specific and/or depends on the particular target gene remains to be investigated.

The strongest evidence for efficient reduction of endogenous hepatic ERα RNA levels *in vivo *was obtained by co-injection of Ad.ERE-luc and advanced non-invasive *in vivo *optical imaging. Administration of Ad.ERE-luc led to readily detectable levels of luciferase activity from day 3 up to day 7 and disappeared at day 10 (data not shown). In agreement with this, the Ad.shERa mediated knock-down effect was present at day three, five, and seven post-injection (Fig 4B). This represents a 4 to 5-day window of expression to determine the phenotypic effects of hepatic shRNA-mediated reduction of mRNA levels.

## Conclusion

We have shown significant repression of hepatic ERα activity in mice utilizing Ad.shERα vectors. In addition, using advanced non-invasive optical imaging technology, the dynamics of the knock-down effect *in vivo *have been demonstrated. Thus, our data confirm that application of shRNA represents a powerful tool for targeted gene silencing. We conclude that Ad-mediated delivery of shERα constructs represents an elegant tool to gain more insight in the role of the hepatic ERα.

## Methods

### Plasmids

Two oligonucleotide pairs (mERα_1395: 5'-gatcccc**gctcctgtttgctcctaac**ttcaagagagttaggagcaaacaggagctttttggaaa-3' and 5'-agcttttccaaaaagctcctgtttgctcctaactctcttgaagttaggagcaaacaggagcggg-3', mERα_1103: 5'-gatcccc**gaatagccctgccttgtcc **ttcaagagaggacaaggcagggctattc tttttggaaa-3' and 5'-agc ttttccaaaaagaatagccctgccttgtcctctcttgaaggacaaggcagggctattcggg) were ordered (Eurogentec, United kingdom). The bold nucleotides correspond to nucleotides 1395–1418 and 1103–1120 of the mRNA mERα sequence (GenBank accession number NM_ 007956). The underlined nucleotides represent a *Bgl*II and a *Hind*III site. These oligo's were annealed and ligated between the *Bgl*II and *Hind*III sites of pSUPER-H1prom [2]. The pSUPER-shERα sequences were verified by restriction and sequence analysis (ABI 3700, LGTC, Leiden).

The H1prom plus or minus shERα were cloned from the pSUPER into the promoter less pAdTrack vector [5] by use of *Xba*I and *Xho*I restriction sites. The Ad.shERα_tandem construct was generated by ligation of H1prom-shERα_1103 between the *Not*I and *Kpn*I sites of pTrack-H1prom- shERα_1395.

The (ERE)_3_TATA-Luc was cloned from pGl_3_-basic as a *Cla*I-blunt/ *Kpn*I fragment in *EcoR*V- and *Kpn*I- digested promoter less Shuttle vector (pShuttle) (He et al. 2509–14). The functionality of this construct was verified by transfection. hERα was cloned from pCMV5 (pCMV5-hERα) [17] as a *BamHI *fragment in the *BglII *digested pShuttle-CMV vector. The pcDNA3.1-mERα expression vector was provided by Larry Jameson [18] and subcloned as a *EcoRI*-blunt fragment in the *EcoRV *digested pShuttle-CMV vector.

### Cell culture

The MXT^+ ^(murine breast cancer) cell line was generously provided by Dr. Bernards. H5V (a murine endothelial cell line derived from heart), EOMA (murine hemangioma-derived micro vascular cell line) and MXT cells were maintained in Dulbecco's modified Eagle's medium (DMEM) (Gibco BRL) supplemented with 10% fetal calf serum, 100 units/ml Penicillin, 100 μg/ml Streptomycin and glutamax (Invitrogen) (Complete DMEM). PERC6 cells [19] were maintained in complete DMEM supplemented with 10 mM MgCl^2+^. For large-scale production of recombinant Ad in PERC6 cells (Crucell, Leiden, he Netherlands), complete DMEM with 2% horse serum (Gibco) was used.

### Luciferase reporter assays

Transient transfections were performed in triplicate in 12-wells plates (1.10^5 ^cells per well) using Lipofectamine (Invitrogen). The effect of shERα on ERα mediated transcription regulation was determined by co-transfecting the cells with 100 ng of reporter construct (ERE)_3_TATA-LUC and 500 ng expression vector pSUPER-shERα or an empty pSUPER control vector together with 100 ng pCMV-LacZ. After 24 hours, the cells were stimulated with complete DMEM containing 10^-9^M Estrogen for an additional 24 hours. The cells were lysed with reporter lyses buffer (Promega) and after centrifugation of 2 min, supernatant was used for determining β-galactosidase normalized luciferase activity by adding 100 μl luciferyl-CoA (Promega) to 20 μl of cell extract in a monolight luminometer (BD Biosciences). β-galactosidase was measured in a 96-well microtiter plate using the β-Galactosidase Enzyme Assay System in reporter lyses buffer (Promega). Absorbance at 450 nm was determined in a microplate reader. Luciferase activities were normalized for transfection efficiency with the β-galactosidase activity and expressed as a percentage relative to expression levels induced by endogenous estrogen receptor (ER). Expression of endogenous ERα in those cells was verified by real time PCR.

### Western blot analysis

Immunoblotting procedures were as described previously [20]. H5V cells seeded in triplicate in 12-wells plate were co-transfected with 20 ng pCMV-mERα and 500 ng expression vector pSUPER-shERα or an empty pSUPER control vector as described above. 28 hours post-transfection, the cells were lysed in 200 μl of RIPA buffer (1% NP40, 0.5% DOC, 0.1% SDS, 50 mM Tris pH 8.0, 150 mM NaCl, 2.5 mM EDTA) containing protease inhibitor (40 ul/ml, Roche). Extracts were cleared by centrifugation (4°C, 14 000 *g*, 5 min), and protein content was determined using the BCA kit (Pierce). Protein samples were denaturated (5 min, 90°C) and separated on SDS/PAGE by use of 8% gradient gels and were transferred to polyvinylidene difluoride (PVDF) membranes (Millipore, Germany). Blots were stained with Ponceau S before blocking to verify equal loading and appropriate protein transfer. Membranes were blocked for 90 min in PBS, pH 7.4, containing 0.05% Tween 20 and 10% milk powder. Thereafter, membranes were incubated for 16 h at 4°C with ab MC20, 1:1000 (mERα rabbit polyclonal antibody, Santa Cruz Biotechnology, CA). After extensive washing with blocking buffer without milk powder or BSA, membranes were incubated for 2 h with horseradish peroxidase-conjugated goat anti-rabbit IgG, 1:5000 (Promega). Membranes were again extensively washed and bound peroxidase conjugates were visualized by enhanced chemiluminescence (ECL, Amersham) on a LumiImager workstation. Additionally, filters were stripped by a 30 min incubation in 100 mM β-mercaptoethanol, 2% SDS, 62.5 mM Tris-HCl pH 6.8 at 50°C, to proceed with the whole procedure as described above. However, now membranes were incubated for 16 h at 4°C with p-38 ab, 1:1000 (N-20, cs-728, rabbit polyclonal antibody, Santa Cruz Biotechnology, CA). Immunoblots were quantified using LUMIANALYST software on a LumiImager (Boehringer-Mannheim).

### Adenoviral vectors

Recombinant adenoviral plasmids were generated by homologous recombination of pAdtrack or pShuttle vectors with pAdEasy1 in BJ5183 cells as described previously [5]. Correct clones were propagated in DH5α cells (Life Technologies). For the generation of the Ad.shERα vectors, Ad.Empty and Ad.ERE-Luc, PERC6 cells were transfected with 4 μg Pac-I-linearized adenoviral construct using LipofectAMINE PLUS (Life Technologies). After 16 hours transfection medium was replaced by growth medium. Transfected cells were harvested at day seven post-transfection and after three freeze-thaw cycles the lysate was used for large-scale production of Ad vectors in PERC6 cells. Virus was purified by double CsCl centrifugation and subsequently dialysed as described previously [21]. Final yields as assessed by plaque assays on 911 cells were approximately 2 × 10^10 ^plaque forming units (pfu)/ml. The control virus (Ad.Empty) carries the green fluorescent protein (GFP) under control of cytomegalovirus promoter (CMV) and contained the H1prom. Ad.shERα_1395 and Ad.shERα_1103 carry GFP under control of CMV and shERα_1395 or shERα_1103 under control of H1prom. Ad. shERα_tandem carries both shERα_1395 and shERα_1103 under control of their own H1prom. Ad.ERE-Luc does not contain CMV-GFP and its functionality was verified *in vitro *and *in vivo.*

### Infection cells

24 hours before transfection, 1.10^5 ^cells per well were seeded into12 wells-plate. Cells were transiently transfected by use of lipofectamine with a total of 450 ng of DNA per well (150 ng of reporter plasmid (ERE)_3_TATA-LUC and 300 ng pCMV-LacZ). After 4 hours cells were infected with either Ad.shERα or control Ad.Empty (MOI 5.000). Additionally, they received 10^-9^M estrogen for 24 hours. Cells were lysed in 300 μl reporter lyses buffer. β-galactosidase and luciferase activity was determined as described above.

### Animals and Ad Injection

The Ethics Committee for Animal Experiments of the Leiden University approved all animal work and the experimental protocols complied with the national guidelines for use of experimental animals. Male C57Bl/6JIco (Charles river, The Netherlands) were given a standard m diet Chow (Hope Farms, Woerden, NL) and housed under standard conditions in conventional cages with free access to water and food.

Recombinant Ad, with a maximum of 4 × 10^9 ^pfu in 200 μl of PBS, were administered by injection into the tail vein of mice at the age of 14 weeks. Within five days post-infusion, mice were sacrificed; liver pieces were removed and immediately deep-frozen in liquid nitrogen and stored at -80°C.

### Pharmacological treatment

The experiment was carried out in 12-wks old C57BL/6 male mice. To prevent sequestration of low doses of Ad.ERE-Luc by liver Kupffer cells, mice were pre-injected with Ad.LacZ (5 × 10^8 ^pfu) 4 hours before administration of 8 × 10^8 ^pfu Ad.ERE-Luc. 17β-estradiol (Sigma, E8875) was dissolved in sesame oil (Sigma). In the dose-response experiment, five days post-injection of Ad.ERE-Luc, 0, 5, 25 and 50 μg/kg 17β-estradiol was injected for 6 hours. Then liver pieces were rapidly dissected and immediately deep-frozen in liquid nitrogen and stored at -80°C for further analysis.

### Bioluminescent reporter imaging

The experiment was carried out in 12 wks old C57BL/6 male mice co-injected with Ad.ERELuc (5 × 10^8 ^pfu) plus either Ad.Empty or Ad.shERα (3 × 10^9 ^pfu). Bioluminescent signals (BLS) were performed at time 0 and at several days after 6 and 24 hours s.c injections of 5 μg/kg 17 β-estradiol with the Xenogen IVIS imaging system (IVIS 100). The living mice were intraperitoneal (ip) injected with the luciferase substrate, luciferin, at a dose of 150 mg/kg body weight approximately 5 minutes before imaging. The mice were anaesthetized with isoflurane/oxygen and placed on the imaging stage. Total photon emission of each animal was acquired for 1 minute. Captured images were then quantified by using the Living Image software (Xenogen Corp, Almeda, CA) and the IGOR software (WaveMetrics Corp, Lake Oswego, OR). BLS from the region of interest (ROI) was expressed using the pseudo colour scale (Red most intense and Blue least intense luminescence) and the data were presented as the cumulative photon counts collected within each ROI. Because layers of tissue may limit photon emission from inner organs, the experiment was repeated. Of these mice the livers were rapidly dissected at day 5, 6 hours after 17β-estradiol administration, verifying the results from the bioluminescent reporter imaging by determining the luciferase activity in liver lysates

### Luciferase enzymatic assay

The liver extracts were prepared by homogenisation with the minibead beater in reporter lyses buffer (Promega), two cycles of freeze-thawing and 2 min. of centrifugation at maximum speed. Supernatants were used for determining protein-normalized luciferase activity by adding 100 μl luciferyl-CoA (Promega) to 20 μl of liver extract in a monolight luminometer (BD Biosciences). Protein content was measured in a 96-well microtiter plate using the BCA protein assay kit (Pierce). Absorbance at 562 nm was determined in a microplate reader.

### Real time quantitative PCR analysis

Total RNA was extracted from liver using TRIzol reagent (Life technologies). Purified RNA was treated with RQ1 RNase-free DNase (Promega, 1 units/2 μg of total RNA) and reverse transcribed with SuperScript II Reverse Transcriptase (Invitrogen) according to the manufacturer's protocol. Quantitative gene expression analysis was performed on an ABI prism7700 Sequence Detection System (Applied Biosystems) using SYBR Green as described earlier (Hoekstra et al. 25448–53). PCR primer sets (Cyclophilline, Fw: AAAAGGAAGACGACGGAGCC Rev: TCGGAGCGCAATATGAAGGT and mERα, Fw: CTAGCAGATAGGGAGCTGGTTCA, Rev: GGAGATTCAAGTCCCCAAAGC) were designed via Primer Express 1.7 software with the manufacturer's default settings (Applied Biosystems) and were validated for amplification efficiency. The absence of genomic DNA contamination in the RNA preparations was confirmed in a separate PCR reaction on total RNA samples that were not reverse transcribed. Cyclophilline was used as a control.

Data Analysis – The significance of differences in relative gene expression numbers C_t _(C_t((Cyclo)_-C_t(target gene)_) measured by real time quantitative PCR was calculated using a two-tailed Student's *t *test. Probability values less than 0.05 were considered significant.

## Abbreviations

Ad5, Human Ad serotype 5; Ad.shERα, Ad mediated expression of shERα; Ad.ERE-Luc, Ad mediated expression of ERE-Luc; ArKO, Aromatase knockout; CCD, Charged coupled device; DMEM, Dulbecco's modified Eagle's medium; ER, Estrogen Receptor; ERE, Estrogen Responsive Element; GFP, Green Fluorescent Protein; Ip, Intraperitoneal; Luc, Luciferase; Pfu, Plaque forming units; S.c, Subcutaneous; siRNA, small interfering RNA; shRNA, short hairpin RNA; shERα, shRNA specific for mouse ERα; Wt, wild type

## Authors' contributions

Y.K carried out the studies described in this paper and drafted the manuscript. F.F participated in the concept of designing shERα. I.Q and C.L. contributed to the imaging experiments shown in Fig 4B. K.WvD participated in the design and coordination of this study and provided expert input for writing the manuscript. All authors read and approved the final manuscript.
